# Epidemic Dysentery of 1849-50: A Paper Read before the Medico Chirurgical Society of Cincinnati

**Published:** 1851-03

**Authors:** 


*Epidemic Dysentery of* 1849–50: *a Paper read before the Medico-Chirurgical Society of Cincinnati.* By John A. Murphy, M. D. With Remarks by the Editor of the Buffalo Medical Journal.

I do not intend, in this paper, to weary the patience of the society, or to abuse its good sense and knowledge of dysentery as a disease, by extending this essay to an immoderate length. Every member of this society has had, in all probability, as much observation of the disease as myself; and possibly each and all may have had better success in its treatment.

I have been impressed with two or three points somewhat new to myself, in the last two years, in the management of this disease, which I have been desirous of presenting to the consideration for some time.

There is, as I conceive, too much eagerness manifested on the part of many in the profession, to make advances and progress (a word, by the way, pregnant with as much evil as good) in every department but that of sound, practical therapeutics. This should not be so, yet I suppose we all must give way, for the time, to the fashion. There is also too much of what may be called physiological practice, on the part of some; while with others, we have the routine practice pursued by those called *experienced.* The latter are much to be preferred. In epidemics, however, they fail; at least, if not failing, the success is comparatively small. In the treatment of dysentery in this city, for the last two years, this certainly has been, in some measure, true. The mercurial practice, in this disease has, I am sure, been inapplicable and unhappy. The hepatic pathology of dysentery is an old one, and, at the present day, an unsound one. The treatment of this disease on its received pathology has proved this true. And here, in passing, I will say, that the depletant and alterative mercurial treatment is receiving, in a great measure, the go-by in all inflammatory affections, at least by a respectable portion of the profession. It is, for one reason, if for no other, too perturbating and depressing. The medical constitution of this whole continent, if not of the world, is asthenic, and has received this impress by the advent of the cholera. That its manner and power of impinging the vital organization has been changed, is true; and that we are justified in attributing this change to the late great epidemic and the one which preceded it, is, I think, too evident to need much demonstration. I know very well that it is unphilosophical to assume a cause, and, in the present case, I hope no one will be inclined to attribute such an assumption to me. That the constitution of diseases was altered by the first epidemic is well attested by the character of those which followed it. It was only succeeding that great epidemic that the attention of the profession was called to the frequent and very general predominance of bowel inflammations in almost all diseases, especially of fevers. During and since the late epidemic, it is a very rare thing indeed to meet with a case of simple or compound bilious remittent fever. But not, however, to indulge in further speculation, I can well and truly say, that we have encountered a form of dysentery, for the last two years, which was severe in its onset, wonderfully rebellious to treatment, accompanied by the entire and perfect depression of all the powers of life. The whole innervation was seriously and powerfully implicated. There can be no doubt, I believe, that the great proximate cause producing this disease, was a modified form of the epidemic virus of cholera. It was modified in its potency and action. As far as I can learn, it was not as severe in this city, and in towns of smaller size, as its predecessor, cholera. And what is somewhat more remarkable, it prevailed in the country with great fatality, and that, too, in many places where but few cases of cholera were observed. The opinion that it was produced by the choleraic poison, gains much plausibility and probability from the fact, that the ordinary causes of epidemic dysentery were not at all in existence; and if they were here and there, but few of those attacked were exposed to them. Cold and dampness have been the great causes originating this disease. Every one here will remember the very warm and dry weather of the past summer. More than all this, epidemic dysentery generally prevails in armies, or in places where large numbers of people are crowded together.

There can be little doubt, I think, held, as to the very prominent cause. The dysentery of the last two years has been so entirely dissimilar to the disease as it ordinarily prevails, that the treatment has been modified and changed in several of its strong and main points. This is the result of my experience. More than this, I am convinced that mercurial and depletant treatment, as ordinarily practised in hepatic dysentery, was wholly inefficient and inapplicable.

The disease, pathologically considered, did not depend, in the great number of cases, on hepatic derangement. Bilious missecretion was not present in the greater part of the cases. I mean by this term, bilious missecretion, a suspension, a change in the character of the bile, or an increased secretion. No pain in right hypochondriac region was complained of. Further, the majority, in the beginning, were what is called dysenteric diarrhœa, the stools being composed, at first, of feculent matter, blood, and some mucus. So much for the hepatic pathology.

The disease consisted essentially of a low type, with a strong tendency to terminate in rapid ulceration and gangrene. If a post mortem examination of the fatal case could have been had, I think that ulceration of the larger part of the colon, with gangrene, would have been found. The ordinary signs and symptoms of severe inflammation of a tonic type, were not present at the beginning of but few cases. I should be much astonished for any one to tell me, that he had treated a case successfully either by decided general or local bleeding. The disease was well marked by all that debility and utter prostraton, which accompanies extensive disease of the glands and mucous lining of the bowels. Instead of the full, strong pulse, hot skin, tenderness on pressure along the track of the co'on, there was the frequent, small, weak pulse, damp, clammy skin, with coldness of the extremities, facies hypocratica, constant tormina and tenesmus and dragging in the lower part of the abdomen, accompanied with urgent desire to go to stool almost constantly. These were a grave class of symptoms, and demanded, as every one well knows, the very opposite of the depletant treatment.

This class of symptoms ushered in the disease in the adult and infant. And here I may say it has dealt severely with children. I have no general data, but I am safe, from my own observation, in saying, that it has formed the largest number in the bill of mortality of children, for the last two years. From the history of many cases, I was not led to believe that the disease had been induced by improper food, or by a change of diet. Too often it succeeded ordinary cases of bilious diarrhœa.

I observed the disease in four different conditions:

1. That following other diseases.

2. That which began as mild dysenteric diarrhœa, and ran rapidly into well-defined.

3. That in which the patient was seized with the disease, in all its severity, at the very onset. In this class of cases, ulceration and gangrene made their appearance early.

4. That form of it when it had become chronic.

This disease was, when present in women, always more severe and resistant to treatment than in men. If in children, it was severe, and as I have before said, in the majority of cases, fatal.

It was of vast trouble when it ensued on other diseases—especially was this the case when it supervened on cholera infantum. Attacking patients convalescing from cholera, the prognosis was bad. I have seen it proceed to ulceration of a chronic character, when it had followed an attack of cholera. This ulceration was very slow and unmanageable. But of this I shall speak further along.

In the second class, or that of dysenteric diarrhœa, or when the stools contained healthy feculent matter, mixed with blood and mucus, and accompanied with tormina and tenesmus, the treatment, if early, was easy and efficient. The stools, in this class were frequent, though tenderness along the colon was not, as a general rule, well marked. Fever was present in very few cases. The dragging in the lower part of the abdomen was generally, and in some few cases was painfully, present. I noticed in all cases a strong disposition to ulceration at an early period. This ulceration was not confined to the rectum, but extended as high up as the head of the colon. The disease similated, in this respect, the severe ulceration of the bowels of 1846, which prevailed along the Ohio and Mississippi rivers. It was this ulceration which carried off so many of the returning and discharged volunteers of the army in Mexico. It prevailed largely in this city at that time. In the Hospital, during that year, I treated a large number of this form of ulceration, and can say that, in many respects, the two diseases were much alike. That ulceration was the disease itself, while this was an early result.

I stated in passing, that there was, as a general rule, no evidence of biliary missecretion in this form. The stools were feculent, mixed with blood and mucus. The three principal features of dysentery, tormina, tenesmus and frequency of discharge, only being present. The stools, so far as the amount of blood and mucus were concerned, were of only secondary importance. In other words, the signs and symptoms seemed only to declare the length which the disease might go, if uncontrolled. The pulse was generally small, frequent and weak—not a pulse at all permiting bleeding. The heat of the surface was not above the ordinary standard, in but few cases. The tongue was moist, covered generally with a whitish fur. There was nothing pathognomonic about it.

This form was much more amenable to treatment than either of the others. The third form of the disease was, by all odds, the severest, most malignant and fatal. The patient was attacked severely, and that with every bad symptom. The tenesmus, tormina and prostration, were great. The stools were of blood and mucus, containing shreds of what seemed to be flesh, mixed with purulent matter. The odor was very fœtid. It was like the cadaveric smell which arises in dissecting rooms, when the weather becomes warm and moist. In one word, it was the odor of mortification. It was an unpromising characteristic. The depression in many of these cases was great, and increased so rapidly that the patients were unable to leave the bed to stool. The tenesmus very often was so great and so continuous, that, in a short time, the muscular contractility of the sphincter ani was paralyzed so as to permit the contents of the bowels to escape involuntarily. The stools, as I have said, were composed entirely of blood and mucus in the beginning, but after the first three or four days, in several cases, were accompanied with green matter, and in other cases, with large lumps of darkish green matter. There was also a large admixture of pus in all of these stools.

The physical sign of severe inflammation, as evidenced by tenderness on pressure along the colon, was not as well marked as could have been expected under such circumstances. There was a tympanitic distention; not, however, severe; owing, I presume, to the extension of the inflammation to the muscular coat. A constant sense of dragging was present, in the majority of cases. It was a very painful symptom.

In women, this dragging sensation was very painful and severe. The disease in women, especially in those of advanced years, or who had borne several children, was malignant, prostrating and unmanageable. This stubbornness and severity arose, I suppose, from the excited sensibility of the uterine system of nerves.

The discharges, as I have before stated, consisted of large quantities of greenish matter. I know of no theory to explain the presence of these discharges, but that they were composed of effused blood, changed in its color by the acid in the bowels. Golding Bird, of London, in an article on cholera infantum, demonstrated, by the microscope, the green discharges of cholera infantum to consist of effused blood, changed by the acids in the bowels.

The supposition that these stools were dependent, in this disease, on morbid secretion of the liver, is to me a very mistaken and fallacious one.

A correct pathology, and a sound therapeutical indication, is very generally, I may say always, proved beautifully and clearly, cæteris paribus, by the treatment.

The fourth form, or the chronic, deserves something more than a passing notice. I view the disease as being chronic, after the third week. The treatment was very difficult and protracted. Ulceration, more or less extensive, was present in all of the cases. The tormina, tenesmus, and blood and mucus, were soon removed, but there continued an irritable state of the bowels, with five or six evacuations in the twenty-four hours.

But let me proceed to pronounce what I have to say on the treatment.

The treatment, then, of this disease, as ordinarily pursued and relied on, failed, or was inapplicable. I mean by this treatment, general and local bleeding, astringents, injections and mercury. The most important of these agents opposed to and cutting short inflammation, blood-letting, was wholly useless, for the reasons I have given in passing. Local bleedings by cups amounted to but little, as the quantity of blood abstracted by the cups, was too small to lessen or influence the inflammation. The benefit resulting from their use was attributable to the counter-irritation. Astringents, as acet. plumbi. tannin, combined with opium, did not act well in the majority of cases. The controlling effect exerted by them was manifested very late, and that in very few cases. I have seen patients, while taking 3 and 4 grs. acet plumbi. with ss. gr. opium every three hours, have just as many stools, and in every respect as painful. Calomel, or blue mass, in conjunction with astringents, acted no better. It was not the disease for calomel as an alterant. Of injections I can speak with no more commendation. Indeed, I am sure I have witnessed the sufferings of the patients increased by enemata; and that too by those composed of simple mucilage and laudanum. Very often the smallest and most soothing injection dexterously administered, would not be retained a minute. The tenesmus was so severe, the irritability of the anus and rectum in other cases, was so great, that a simple suppository of extract opii would be expelled the instant it was introduced.

My precept of practice in regard to the use of enemas, was founded on the severity of the tenesmus: if it was great, if almost constantly present, and if accompanied with small, bloody mucus and puerlent stools, with the odor of mortification, I did not order them. I did not order them for I soon found they would not be retained, and often aggravated the tenesmus. I have used a great many articles in enema, as acet. plumbi. tannin, tinc. opii, bals. copaibæ, mucilage, &c. In several cases, enemas of cold water were not retained for any length of time, and did not seem to have any good effect.

The idea of salivating patients under such circumstances, is a very bad one: the attempt to do so is very profitless and oftener impossible. The treatment is not as rational to me as that of salivating a patient with typhoid fever, and that I hope every one here believes is utterly bad, unsound and absolutely the signed warrant of death to the patient.

To ptyalize when we are ignorant of what else to do, is only worthy of a medical man of the mediæval ages, and as such should be banished and cast out from all sound practice. There are two conditions of the economy in which we can not obtain the constitutional effect of mercury; that of exalted, and that of depressed action. As an illustration of the latter, let each and all recur to few instances of salivation in those cases of cholera where such immense quantities of calomel was given. Drs. Delamater and Ackley of Cleveland state that but few cases were salivated of all those who were treated after Ayres's plan, the most successful, by the way, of all others. The calomel, they say, was found in large quantities lining the mucus membrane of the stomach and bowels of those who died. It acted in some way on the secretions of the bowels, which were highly acid from the lack of a proper proportion of alkalies in the blood; and frequently I have no doubt, dysentery was induced by this cause.

In the great number of cases, the debility was so great, that salivation could not be induced, and if it was brought about, it was just half past the "eleventh hour." Calomel was not needed but sparingly, much less its constitutional effect. It acted as an irritant; it added to the already great irritation of the constitution; it detracted from the already too small quantity of fibrine in the blood; it stimulated the liver to morbid action: in one word, it produced mercurial bile—a term which every one will understand.

Here let me stop to say, that I have no specific treatment of dysentery—no one or many articles combined, which cured all cases. But this much I can say, that the prescription which I shall give shortly effectuated the removal of the disease more safely, more speedily, and more pleasantly to the patient and doctor, than any thing else I tried.

It must be remembered always, when speaking of remedies, no matter how potent and how various their power in curing, that there are a certain number of cases of every disease, be it epidemic, ideopathic or sporadic, which are what Sir Gilbert Blane calls "determinedly fatal."

My favorite prescription was oil sweet almonds ʒij. to ℥ss., pulv. accaciæ ℥j, laurel water ℥ss., sugar ʒj., sulph. morphine gr. j. to gr. jss., water ℥iij., of which a half to a full tablespoonful was to be taken every three hours.

Some care is to be had in making this emulsion. The oil of almonds and the morphine is to be rubbed up well with gum accaciæ, and then the laurel and common water is to be added. It is especially necessary to have the oil almonds fresh and free from rancidity, while it is well blended with the g. accaciæ. When the discharges were frequent, I have often, and that with good effect, added ℈j. acet. plumbi. Generally, however, I have given the original prescription.

This prescription was first used by my friend Dr. Unzicker. My friend, Dr. David Judkins, first reported to me its genial effect last year, since which time I have used it with the best results. The acetas plumbi did not belong to the original prescription.

It constituted, however, my main medical treatment in the disease. It very soon relieved the tormina, tenesmus and frequency of stools. It brought about a cure in from ten days to two weeks. During its administration, I occasionally gave x. or xv. grs. pill mass hydg., with ext. hyoscyam, in divided doses to counteract the restraining action of the opium on the secretions generally. At other times I gave one or two grs. calomel, for the same purpose. In conjunction with this, I resorted early to stimulants.

If the disease was of the severe character, I ordered brandy, beef tea, chicken tea, and so on. If milder, I gave port wine. I prescribe stimulants irrespective of fever. Iced gum arabic water was given to allay the thirst.

Not in one case could I perceive any bad results from the stimulants. The local and constitutional irritation was so great that stimulants were essentially necessary.. The doctrine of Travers, in his monograph on constitutional irritation is certainly correct, when he says, "debility is the basis of morbid irritation, and those causes of debility which operate with the greatest force and directness most invariably aggravate the state of irritation."

That questio vexata, the modus operandi of the prescription, may be raised upon me here.

The great efficacy is owing to the peculiar combination. In addition to the sedative and anodyne power of the morphine, we have that also of the almond oil and laurel water.

The morphine did not control the disease by itself. Passing over this point, I can repeat what I have before said, that I observed the most admirable results from it.

In chronic dysentery I continued its use until the tormina and tenesmus were removed. To heal the ulcers, I alternated the syrup ferri iodide, in xv. grs. doses, three times a day, with a pill of nitrat. argent $\frac{1}{2}$ gr., sulph. morphine $\frac{1}{8}$ gr., four times a day. I have treated a large number of cases of this disease in the last two years, and have lost very few. Here I may say, that the treatment of dysentery, by the regular profession in this city, has been very successful.

I have to regret my inability to present the results of a post mortem examination. One word in conclusion and I am done. I have described the disease as I saw it, and have given the treatment which, after much anxious trial, I found the most successful.*

*Remarks by the Editor of the Buffalo Medical Journal.*—The considerations presented in the foregoing essay relate to a highly interesting and important practical subject, and are intrinsically valuable. It is gratifying to observe that the hepatic pathology of dysentery is receiving its just deserts in the estimation of the intelligent portion of the profession. What can be more gratuitous than to hold the liver responsible for the phenomena due to a phlogosis of the mucous tunic of the large intestine? All the data upon which this hypothesis is based are assumed, not only without proof, but in direct opposition to plain facts. The hepatic practitioner tells us that the philosophy of dysentery involves, first and chiefly, suppression of the secretion of bile; next, in the order of sequence, congestion of the portal circle, and from these morbid conditions combined, the intestinal symptoms pertaining to the disease derive their origin. Now, the point of departure for this imaginary train of events is wholly supposititious. It may be doubted if the secretion of bile be more affected in this disease than in other disorders into the pathology of which it is not supposed to enter. And if it were, why should such consequences be expected to follow? Is not the biliary secretion suppressed totally in some cases of icterus, without dysenteric, or any symptoms of intestinal disease being present. "Congestion of the portal circle," too, an expression so often quoted, so convenient and satisfying to the minds of some—what are the positive evidences that such a morbid condition plays an important part, or even that it is present at all in dysentery? But admitting the portal system to be congested, what right

* An interesting discussion occasioned by the reading of the foregoing essay is omitted for want of space.

have we to infer that any of the symptoms of dysentery are due to that source? We know that the portal system is congested in cirrhosis of the liver to a degree causing mechanical transudation of the serosity of the blood through the coats of the vessels into the peritoneal cavity; but we do not meet with dysentery in that connection.

Place against these gratuitous assumptions the positive facts so easily developed if practitioners will take the trouble to examine the mucous tunic of the large intestine in a few fatal cases of dysentery—the increased vascularity, thickening, softening, ulcerations extending, more or less, from the outlet of the rectum to the ileum, or even higher in the intestinal tube—and the hepatico-congestive theory will appear puerile enough to all except those in whose pathological notions the "liver (to quote the language of our friend Dr. T. S. Bell) is the central sun around which all the other organs of the body revolve as so many satellites."

The factitious influence attributed to the Liver in the production of this, as well as of many other diseases, has led to therapeutical errors which must be abandoned, or materially modified, just in proportion as the vicious pathological ideas upon which they rest are supplanted by those more in accordance with the present condition of science. The practitioner who sees in the phenomena of dysentery suppression of bile and portal congestion as the prime and essential morbid conditions—the first links in the chain of causation—will be timid in the use of opiates for fear of increasing the congestion still more, and diminishing, if possible, in a greater degree the biliary secretion; and, with the notions which have been current respecting the specific action of mercurials, he will be likely to employ calomel in excessive doses to 'act upon the liver, and promote free circulation through the portal circle.' The effect, thus, of the hepatic pathology, here as in other instances, is positively and negatively bad. It stands in the way of remedies calculated to relieve painful symptoms, and abate the severity of the disease, and it encourages the injudicious use of a valuable remedy, the abuse of which has done not a little to bring the practice of medicine into discredit.

These remarks have been suggested by reading the article which precedes this, copied from the Western Lancet. The subject of dysentery opens a wide field for remark. We have noticed only one of the many topics which a full discussion of the subject would embrace; but we have no design of engaging in such a discussion at this time. We will, however, add a few observations.

There are few diseases which present such wide extremes as respects degree, as well as such variations in important symptoms, as dysentery. In its sporadic and epidemic forms it is hardly the same disease, the points of difference preponderating so much over those of agreement. In ordinary sporadic dysentery the affection seems to be purely a local affection, not extending even over the whole tract of the large intestine, and sometimes confined to the rectum. Such cases almost uniformly recover, and efficient treatment is not called for. In epidemic dysentery, on the other hand, the local inflammation is more extensive, affecting the whole of the large, and perhaps more or less of the small intestine. It is easy to understand that the gravity of the symptoms due to an inflammation of the external surface must be commensurate with the extent of surface inflamed; and it is obvious that the same rule should hold good with respect to an internal surface. An area of the cutaneous tegument equal to that of the mucous tunic of the large intestine expanded into a plane, if affected, for example, with erysipelas, would by no means be a trivial affection. Here then, is one cause of disparity.

But another and probably not less important cause involves the immediate action of a morbific principle upon the system. Epidemic dysentery is a zymotic affection, of which the inflammation of the intestine is but a local expression—an effect, or a complication. Dr. Ramsay's views on this point we doubt not to be correct. Hence, the gravity of the symptoms will be affected by the degree of those inappreciable changes appertaining to the system which are the proximate effects of the action of the epidemic poison. Hence the variations in different seasons, and places, as well as in different individuals due to the special cause, and the circumstances appertaining to its action upon the system, irrespective of the intestinal affection.

The intestinal inflammation in many cases of epidemic dysentery, bears to the whole disease a relation somewhat similar to that existing between the pneumonitis which occurs as a complication of Typhus, and the febrile disease. We might, with as much propriety, say that the pneumonitis, in the latter case, constituted the disease, as in the former case, to overlook a morbid state ulterior to the local affection.

If these views be correct, a routine uniformity of treatment for this disease is, in the abstract, an absurdity, and, in its consequences, can hardly fail to be, in a certain proportion of cases, productive of a degree of harm proportionate to its potency for evil as well as good. That pathology which recognises "but one organ, the liver,—one disorder, the biliary,—and one remedy, calomel," however it may commend itself for its simplicity and convenience, will prove, in its practical results, to be far from an innocent vagary.

It is at once obvious that a rational treatment comprehends measures appropriate to the local and general morbid conditions, and adapted to meet the variations incident to time, place, and individual cases. This mode of expression refers to generalities only, but we cannot here amplify upon this point sufficiently to consider particulars. Suffice it to say, that in fulfilling the various and varying indications of treatment which belong to this disease, opiates, astringents, tonics, stimulants, mercurials, etc., may all be brought into requistion; and, in the management of this disease, as in other diseases, that practitioner will be the most successful who is best qualified to appreciate in each case the particular objects which it is desirable to fulfil by remedies, and who, in connection with an abundant personal experience, and an extended acquaintance with the experience of others, can bring the best judgment and tact in applying means to effect these objects.

